# The Impact of Social Isolation and Loneliness on Health Outcomes After Traumatic Brain Injury: A Scoping Review

**DOI:** 10.1093/geroni/igaf122.3124

**Published:** 2025-12-31

**Authors:** Wonkyung (Kelly) Jung, Tess Chernauskas, Kuan-Ching Wu

**Affiliations:** Boston College, Chestnut Hill, Massachusetts, United States; Boston College, Chestnut Hill, Massachusetts, United States; Emory University, Atlanta, Georgia, United States

## Abstract

Traumatic brain injury (TBI) often leads to social isolation and loneliness, which can significantly affect an individual’s mental, physical, and social well-being, particularly in older adults. Despite these serious consequences, the extent to which social isolation and loneliness impact health outcomes after TBI has not been thoroughly explored. This scoping review aims to map the literature on the effects of social isolation and loneliness on health outcomes following TBI. By examining studies across a range of health domains, we seek to better understand the association between these psychosocial factors and recovery trajectories in TBI populations. A scoping review was conducted following the Preferred Reporting Items for Systematic Reviews and Meta-Analyses (PRISMA) extension for Scoping Reviews. A comprehensive search across PUBMED, CINAHL, EMBASE, PsycINFO, and Web of Science identified studies published between 2014 and 2024. A total of 1,250 studies were screened after duplicates were removed, and eight studies met predefined inclusion criteria. The results indicate that social isolation and loneliness after TBI are associated with worse health outcomes, including higher rates of depression, PTSD, slower recovery, and reduced functional abilities. These psychosocial factors also contribute to increased morbidity, mortality, and reduced quality of life. Social isolation and loneliness pose significant barriers to recovery after TBI. Addressing these factors through targeted interventions may improve both short- and long-term health outcomes. Further research is needed to explore underlying mechanisms and develop effective strategies to mitigate their negative effects in TBI populations.

